# Choosing a Physician

**Published:** 1877-04

**Authors:** 


Choosing a Physician.—"To choose a physician," as Lady Mountcashel has well remarked, "one should be half a physician one's self; but as this is not the case with many, the best plan which a mother of a family can adopt is to select a man whose education has been suitable to his profession, whose habits of life are such as prove that he continues to acquire both practical and theoretical knowledge, who is neither a bigot in old opinions nor an enthusiast in new; and, for many reasons, not the fashionable doctor of the day. A little attention in making the necessary inquiries will suffice to ascertain the requisites here specified; to which should be added what are usually found in medical men of real worth, those qualities which should serve to render him an agreeable companion; for the family physician should always be the family friend."

